# Characterization of Altered Oropharyngeal Microbiota in Hospitalized Patients With Mild SARS-CoV-2 Infection

**DOI:** 10.3389/fcimb.2022.824578

**Published:** 2022-03-15

**Authors:** Yong-Lin Shi, Mao-Zhang He, Mao-Zhen Han, Hong-Ya Gui, Peng Wang, Jun-Ling Yu, Ying-Lu Ge, Yong Sun, Sheng-Hai Huang

**Affiliations:** ^1^ Department of Microbiology, The Key Laboratory of Microbiology and Parasitology of Anhui Province, The Key Laboratory of Zoonoses of High Institutions in Anhui, School of Basic Medical Sciences, Anhui Medical University, Hefei, China; ^2^ School of Life Sciences, Anhui Medical University, Hefei, China; ^3^ Anhui Provincial Centers for Disease Control and Prevention, Hefei, China

**Keywords:** oral swab specimens, nanopore sequencing, full-length 16S rRNA, microbial analysis, SARS-CoV-2 patients, oral microbiota

## Abstract

Coronavirus disease 2019 (COVID-19) remains a serious emerging global health problem, and little is known about the role of oropharynx commensal microbes in infection susceptibility and severity. Here, we present the oropharyngeal microbiota characteristics identified by full-length 16S rRNA gene sequencing through the NANOPORE platform of oropharynx swab specimens from 10 mild COVID-19 patients and 10 healthy controls. Our results revealed a distinct oropharyngeal microbiota composition in mild COVID-19 patients, characterized by enrichment of opportunistic pathogens such as *Peptostreptococcus anaerobius* and *Pseudomonas stutzeri* and depletion of *Sphingomonas yabuuchiae*, *Agrobacterium sullae*, and *Pseudomonas veronii*. Based on the relative abundance of the oropharyngeal microbiota at the species level, we built a microbial classifier to distinguish COVID-19 patients from healthy controls, in which *P. veronii*, *Pseudomonas fragi*, and *S. yabuuchiae* were identified as the most prominent signatures for their depletion in the COVID-19 group. Several members of the genus *Campylobacter*, especially *Campylobacter fetus* and *Campylobacter rectus*, which were highly enriched in COVID-19 patients with higher severe acute respiratory syndrome coronavirus 2 (SARS-CoV-2) viral load and showed a significant correlation with disease status and several routine clinical blood indicators, indicate that several bacteria may transform into opportunistic pathogen in COVID-19 patients when facing the challenges of viral infection. We also found the diver taxa *Streptococcus anginosus* and *Streptococcus alactolyticus* in the network of disease patients, suggesting that these oropharynx microbiota alterations may impact COVID-19 severity by influencing the microbial association patterns. In conclusion, the low sample size of SARS-CoV-2 infection patients (n = 10) here makes these results tentative; however, we have provided the overall characterization that oropharyngeal microbiota alterations and microbial correlation patterns were associated with COVID-19 severity in Anhui Province.

## Introduction

Coronavirus disease 2019 (COVID-19) was recognized as a global pandemic established at the end of 2019 and is an infectious disease caused by the SARS-COV-2 virus (Zhou et al., 2020; Zhu et al., 2020). Globally, as of October 18, 2021, there have been more than 240,260,449 confirmed cases of COVID-19, including 4,890,424 deaths; nonetheless, this number is still increasing rapidly (Coronavirus W., 2021). COVID-19 ranges from mild to severe; however, the majority of infected individuals show moderate or mild symptoms and eventually recover from COVID-19. Hence, it becomes exigent to detect potentially infected people quickly and early, particularly those with asymptomatic infections, which is a prerequisite to solve the rapid spread of this disease. Presently, the gold standard for the diagnosis of SARS-CoV-2 infection is the detection of nucleic acids in upper respiratory tract samples by reverse transcriptase–PCR (RT–PCR) (Wolfel et al., 2020). However, due to virus variation, sampling errors, low virus titers (Su et al., 2020), and other reasons, the false-negative rate of RT–PCR is at least 20% (Cheng et al., 2020). Therefore, it is imperative to find a more accurate and efficient diagnosis method.

There is already a consensus that the host indigenous microbiota plays a pivotal role in modulating human health by forging the immune system and maintaining homeostasis, and it is well known that microbiota dysbiosis or imbalance is closely related to various diseases. Many previous studies have delineated the dysbacteriosis and disordered functionality of gut and respiratory tract microbiota in COVID-19 (Zuo et al., 2020; Yeoh et al., 2021; Zhang et al., 2021; Zuo et al., 2021). Although a handful of studies have depicted oral microbial features in COVID-19 patients or after recovery (Iebba et al., 2021; Ren et al., 2021; Wu et al., 2021), the characterization of oral microbes conducted by full-length 16S rRNA sequencing in mild COVID-19 has not been reported. In fact, the oral cavity is documented as the second-largest microbiota in the human body and plays an important role in the pathogenesis of infectious diseases. Previous studies have reported that microbes that interact *via* the oral-lung axis can affect the outcome of many infectious diseases by regulating host mucosal immunity (Mammen et al., 2020; Li et al., 2019; Bao et al., 2020). In the current study, some reports hinted that the oral cavity can be used as a potential reservoir not only for SARS-CoV-2 but also for a disturbance microbiota with lung pathogenic potential (Xiang et al., 2020). Thus, characterizing the oral microbiota structure when SARS-CoV-2 is present may identify physiological markers for the potential risk in terms of disease severity and therapeutic strategies. Moreover, full-length 16S rRNA gene sequencing will accelerate the speed and promote the resolution of detecting potential pathogenic bacteria accompanied by SARS-CoV-2 infection. In the present research, we characterized the influence of SARS-CoV-2 on the composition of the oral microbiota in COVID-19 patients.

## Methods

### Sampling Location and Collection

This prospective study involved 10 patients (five men and five women) with COVID-19 hospitalized with laboratory-confirmed SARS-CoV-2 infection patients (LCP) were admitted to the Anqing Municipal Hospital of Anhui Medical University and Affiliated Fuyang Hospital of Anhui Medical University from February 18 to 26, 2020, and 10 healthy individuals [healthy controls (HCs)] were admitted to the First Affiliated Hospital of Anhui Medical University from February 15 to 28, 2020. Accordingly, the 20 individuals incorporated into the study were recorded without using antibiotics within 2 months. SARS-CoV-2 infection was confirmed by 2 consecutive RT–PCR tests targeting the ORFlab and N genes. HCs were individuals with no past medical history or history of antibiotic intake in the past 3 months recruited *via* advertisement from the general population who tested negative for SARS-CoV-2. All subjects were recruited between February and March 2020. The severity of COVID-19 infection was categorized as mild according to Ct. value. All patients provided informed consent to participate in this study. Throat swab samples from patients with COVID-19 were collected serially 2 to 3 times per week until discharge (first sampled swab samples were selected for subsequent experiments). Serum samples were collected after routine diagnostic testing on patients admitted to the hospital who subsequently tested positive for SARS-CoV-2 *via* PCR. This study was conducted in accordance with the Declaration of Anhui Medical University.

### Detection of SARS-CoV-2 Viral Load

SARS-CoV-2 detection was performed on the CFX96™ Real-Time PCR Detection System (Bio-Rad, Hercules, CA, USA) using the NeoPlex™ COVID-19 Detection Kit (GeneMatrix, Seongnam, South Korea) targeting viral ORFlab and N genes and the housekeeping gene of β-actin as an internal control, following the manufacturer’s instructions.

### DNA Extraction, 16S rRNA Gene Amplification, and Single-Molecule Real-Time Sequencing

Bacterial DNA was extracted from the throat swabs by using a QIAamp DNA Microbiome Kit (QIAGEN, Hilden, Germany) according to the manufacturer’s instructions and eluted with nuclease-free water. The DNA was quantified by using a Qubit^®^ dsDNA BR Assay (Qubit^®^ 2.0 Invitrogen Life Technologies, Waltham, MA, USA), and the quality was checked by running an aliquot on a 1% agarose gel stained with ethidium bromide, stored at −80°C, and used as needed. When the 16S rDNA PCR result was positive, sequencing libraries were prepared from the PCR products using the Rapid Barcoding Sequencing Kit (SQK-RBK004; ONT, Oxford, UK). The input DNA was end-repaired and A-tailed using the Ultra II End Prep Enzyme (New England Biolabs [NEB], Hertfordshire, UK) incubated at 20°C for 5 min and 65°C for 5 min. The end-prepared DNA was purified with AMPure XP (Beckman Coulter, High Wycombe, UK), and the DNA was eluted in nuclease-free water followed by ligation with a 1D adapter using Blunt/TA Ligase Master Mix (NEB) at room temperature for 10 min. 1D adapter DNA purification was achieved with Adapter Binding Buffer (ONT) using a magnetic stand, and the DNA library was eluted with elution buffer (ONT). The presequencing mix was loaded onto an R9.5 flow cell (FLO-MIN107) in a mix of running buffer with fuel mix and library loading buffer (ONT). Finally, sequencing was performed for 2 or 3 h, and base calling was performed using MinKNOW software.

### Taxa Feature Selection With the Random Forest Model and Microbial Correlation Network

To determine the species that could be used to distinguish healthy and COVID-19 patients, a random forest model was built with modified settings (mtry = 39, ntree = 600). The optimal number of species was determined by 10-fold cross-validation using the *rfcv* function provided by the random forest package. The most highly discriminating species were identified by importance values characterized by “MeanDecreaseAccuracy” parameters. The microbial correlation networks were constructed using SparCC (Friedman and Alm, 2012). Significant interactions were determined by the bootstrapped results (N = 100) using the script PseudoPvals in SparCC. Significant correlations with absolute sparse correlations ≥ 0.3 and p-value ≤0.05 were visualized using the igraph package in R.

### Statistical Analysis

The Bray–Curtis distances were calculated based on the taxonomic species-level data using the R package *vegan*. Principal coordinate analysis (PCoA) was performed to highlight the discrepancy of the phylogenetic compositions of gut microbiota between the LCP and HC groups using the *ade4* package in R. An analysis of similarities (ANOSIM) was used to assess the differences in gut microbiota among the groups using the R package *vegan*. An R value close to 0 represents no significant difference between inter- and intragroup differentiations. An R value close to 1 indicates that intergroup differentiation was greater than intragroup differences. p ≤ 0.05 reflects a statistical significance. For comparison analysis, a Wilcoxon rank-sum test was used for the comparison of gut microbial composition that did not fit the normal distribution between the two groups. Raw p-values were adjusted for multiple testing using the Benjamini–Hochberg method at a false discovery rate (FDR) of 0.05 (Benjamini and Hochberg, 1995). Spearman’s rank correlation was used to assess the relationship between differential bacterial taxa and SARS-CoV-2 Ct. value, followed by routine blood indicators (RBI) after adjusting covariates age and sex. Multiple testing was adjusted using the Benjamini–Hochberg method. Scatter plots and heatmaps were plotted using the ggpubr and ggplot2 packages in R software.

## Results

### Overall Characterization of the Bacterial Composition in the Study Subjects

**Figure 1:**
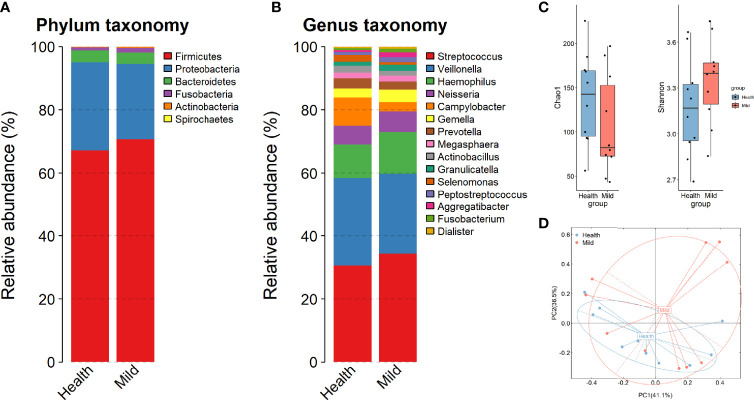
Changes in microbial composition in mild COVID-19 patients compared to healthy controls. **(A, B)** Relative abundance of the top bacteria at the phylum and genus levels. **(C)** Chao1 diversity and Shannon biodiversity plot between healthy and SARS-CoV-2 (mild) individuals. **(D)** Bray–Curtis analysis of bacterial community composition diversity between healthy and SARS-CoV-2 samples.

To depict the microbial profiling in those individuals, we sequenced the full-length bacterial 16S rRNA gene by nanopore sequencing. As expected, our data exhibited a divergent microbiota composition between the LCP and HC groups in throat swab samples to a certain degree. The three most abundant phyla were Firmicutes, Proteobacteria, and Bacteroidetes (accounting for 96.1% and 98.54 in each group), which was in accordance with previous research (Wu et al., 2021) (**Figure 1A**). At the genus level, we found that three predominant bacteria, *Streptococcus*, *Veillonella*, and *Haemophilus*, together accounted for 60% on average in both groups (**Figure 1B**). The alpha- and beta-diversity metrics between oral bacteria from the HCs and patients with mild conditions during hospitalization were compared. The Chao1 and Shannon indices of each sample were used to evaluate the species richness and diversity. The Mann–Whitney test was applied to evaluate any significant difference in the bacterial richness and diversity between the LCP and HC samples. We found a decreased Chao1 index and increased Shannon diversity in the LCP group compared to the HC group, although there was no significant difference between the two groups of samples (p > 0.05) (**Figure 1C**). The overall bacterial structure difference was further verified by the PCoA plot and permutational multivariate analysis of variance (PERMANOVA) with the Bray–Curtis distance calculated at the taxonomic species level. The bacterial composition of the oral samples in LCP and HCs diverged from each other along the first and second axes, in which 77.6% of the total variance was explained, indicating that the largest source of variation was the disease status (PERMANOVA, R^2^ = 0.125, p < 0.05) (**Figure 1D**). These results hinted that a clear separation of the bacterial structure was gained after SARS-CoV-2 infection in throats.

### Changes in the Throat Microbiota Profile in COVID-19 Patients

**Figure 2:**
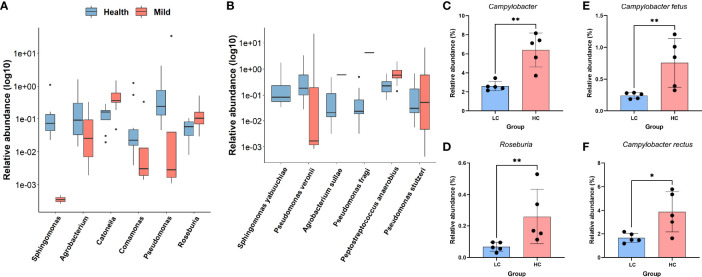
Differentially abundant genera or species in COVID-19 patients and healthy controls. **(A)** Log10-transformed relative abundance of significantly different taxa at the genus level. **(B)** Log10-transformed relative abundance of significantly different taxa at the species level. **(C–F)** Relative expression levels of selected genera **(C, D)** and species **(E, F)** differing between lower viral titers and higher viral titers. **p < 0.01; *p < 0.05; Wilcoxon rank-sum test.

To further identify which bacteria were altered in the oropharyngeal region associated with COVID-19, the Wilcoxon rank-sum test was employed in the case of non-parametric data. Twelve taxa were identified as significantly different in the oral microbiota between the LCP and HC groups. Specifically, significant increases in *Catonella* and *Roseburia* were observed at the genus level (**Figure 2A**), as well as *Peptostreptococcus anaerobius* and *Pseudomonas stutzeri* were observed in the SARS-CoV-2-infected patients compared to the HCs (**Figure 2B**). In contrast, *Sphingomonas*, *Agrobacterium*, *Comamonas*, and *Pseudomonas* at the genus level (**Figure 2A**), along with *Sphingomonas yabuuchiae*, *Pseudomonas veronii*, *Agrobacterium sullae*, and *Pseudomonas fragi*, were enriched in the HCs compared to patients (**Figure 2B**). In addition, to investigate the potential influence of sex and SARS-CoV-2 viral load on bacterial compositions in patients, a similar comparison was performed between men and women, with higher viral load (based on the value of Ct. by qPCR) and lower viral load within the LCP group. No significant differential sex-biased alpha-diversity indices of bacterial taxa were found. Nevertheless, the results of alpha-diversity analysis showed that individuals with higher SARS-CoV-2 viral load exhibited lower bacterial richness and diversity, and beta diversity results showed a significant distinction of oral microbial communities between higher viral load patients as compared to lower ones (**Figure S1**). In addition, bacterial taxa involving *Campylobacter* and *Roseburia* at the genus level (**Figures 2C, D**), plus *Campylobacter fetus* and *Campylobacter rectus* at the species level, were diminished in patients with higher SARS-CoV-2 viral load (**Figures 2E, F**).

### Microbial Correlation Networks and Relationships Between SARS-CoV-2 Virus Load, Routine Blood Indicators, and Bacterial Species

**Figure 3:**
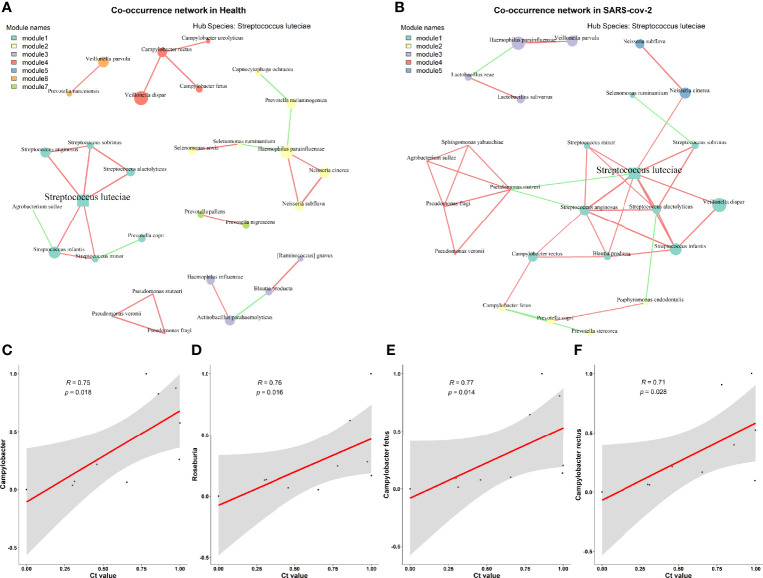
Microbial correlation network between healthy and mild SARS-CoV-2 patients. **(A)** Healthy controls. **(B)** SARS-CoV-2 patient. **(C–F)** Relationships between Ct. values and the differential taxa identified between higher and lower SARS-CoV-2 viral titer groups at the genus level **(C, D)** and species level **(E, F)**.

Considering that gut bacteria are complex in both structure and function, this complexity can be well represented and modeled as networks. Network methods can be applied to microbiota studies to model the co-occurrence of bacteria, find microbial relationships essential for community assembly or stability, and deduce the influence of various associations on host health. To compare the microbial communities of the two groups at the network level, we constructed a species-level microbial correlation network for each group using the package SparCC (Friedman and Alm, 2012) (sparse correlations for compositional data). We found that the microbial correlation network of the LCP group had quite different structures compared to the HC group (**Figures 3A, B**). More precisely, it has fewer nodes but higher edges and a higher average degree and betweenness centrality (**Supplementary Table 1**). These results indicate that the overall microbial correlations in the LCP group are much stronger than those in the control group. Furthermore, to analyze network patterns in detail, NetShift (Kuntal et al., 2019) was leveraged to identify potentially important “driver” species responsible for the change in microbial correlations. According to their neighbor shift (NESH) scores and betweenness centrality (BC) measurements, the top driver species were *Streptococcus anginosus*, *Streptococcus alactolyticus*, and *P. stutzeri* in the LCP group (**Figure S2**). These results hinted that certain bacterial taxa (e.g., *S. anginosus*, *S. alactolyticus*, and *P. stutzeri* excluding hub species *Streptococcus luteciae* in both groups) could play an important role in driving the changes in microbial correlations in subjects with SARS-CoV-2 infection.

**Figure 4:**
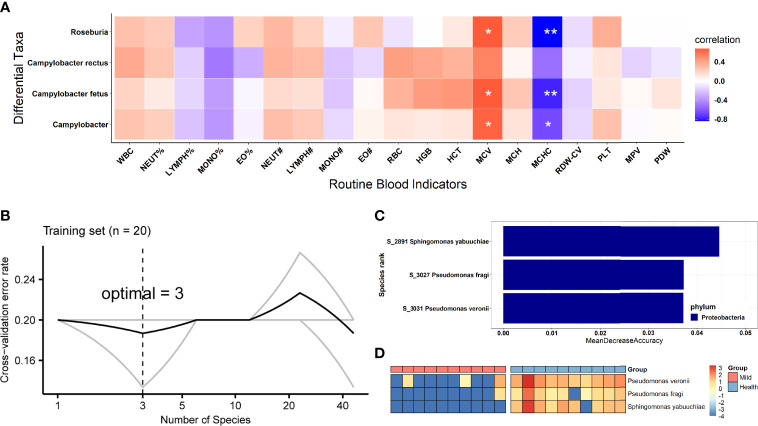
Biological relevance between the differential oral bacteria and clinical RBI and the identification of biomarkers based on oral microbiota for SARS-CoV-2 patient diagnosis. **(A)** Heatmap of Spearman’s rank correlation coefficients between differential oral bacteria based on viral load and clinical blood parameters. Statistically significant associations are shown with asterisks, as follows: ^*^p < 0.05, ^**^p < 0.01. **(B)** The top 3 biomarker bacterial species were identified by applying a random forest classifier of their relative abundances. **(C)** Biomarker taxa are ranked in descending order of importance to the accuracy of the model. **(D)** Heatmap showing the relative abundances of the top 3 disease-predictive biomarker bacterial taxa. WBC, white blood cells; NEUT%, neutrophilic granulocyte percentage; LYMPH%, lymphocyte percentage; MONO%, monocyte percentage; EO%, eosinophil percentage; NEUT#, absolute neutrophil count; LYMPH#, absolute lymphocyte count; MONO#, absolute monocyte count; EO#, absolute eosinophil count; RBC, red blood cells; HGB, hemoglobin; HCT, hematocrit; MCV, mean corpuscular volume; MCH, mean corpuscular hemoglobin; MCHC, mean corpuscular hemoglobin concentration; RDW-CV, red cell distribution width; PLT, platelet count; MPV, mean platelet volume; PDW, platelet distribution width; RBI, routine blood indicators.

Additionally, we examined whether the SARS-CoV-2 viral load was associated with increased or decreased oral bacterial species in higher SARS-CoV-2 infection patients. At the genus level, *Campylobacter* and *Roseburia* were both identified as significantly anti-correlated with the viral load of SARS-CoV-2 (**Figures 3C, D**). Next, *C. fetus* and *C. rectus* were negatively associated with the viral load of SARS-CoV-2 at the species level (**Figures 3E, F**). Meanwhile, the genera *Campylobacter* and *Roseburia* and species *C. fetus* were positively associated with the blood level of mean corpuscular volume (MCV) but negatively correlated with the mean corpuscular hemoglobin concentration (MCHC) level (**Figure 4A**).

### Identification of COVID-19-Specific Important Bacterial Signature Based on the Oral Microbiota

To explore which oral microbiota can be used as an important signature in SARS-CoV-2-infected patients, we constructed a random forest model between 10 HCs and 10 LCP based on species abundance (finer level). Initially, the model constructed using the microbiota at the species level showed the best predictive accuracy of 80% (e.g., the out-of-bag error rate was 20%) among all taxonomic levels, and species that could accurately identify differences between both groups were selected as the optimal marker set through fivefold cross-validation in the random forest model. This result indicated that the optimal number of bacterial signatures (species) was three (**Figure 4B**). Regarding these 3 species, the importance index (“MeanDecreaseAccuracy”) is shown in **Figure 4C**. We detected that all 3 species (*P. veronii*, *P. fragi*, and *S. yabuuchiae*) decreased in abundance in infected patients (**Figure 4D**). Furthermore, the three species identified as having a COVID-19 signature by the random forest method were consistent with those detected by the non-parametric Wilcoxon test. After the three species (disease biomarkers) were identified, we constructed a new random forest model as a disease classifier using these species. The out-of-bag error rate of the new model was 13.33%, which was higher than that of the model constructed using all microbiota members at the species level.

## Discussion

To date, COVID-19 has been proven to be a pivotal threat to our lives. Recently, several lines of evidence have disclosed that the oral cavity should be noticed for its preferential route for SARS-CoV-2 entry or transmission (Netz and Eaton, 2020; Jiao et al., 2021). The oral commensal microbiome has been documented to provide a range of beneficial properties to the host. Previous studies have reported that several of the most significant contributions of these microbes are to boost metabolism, improve digestive health, and protect against invading pathogens. Furthermore, the coordinated crosstalk between commensal microbes and different body systems is essential for the functioning of the immune system (Freire et al., 2021). Here, our study attempts to leverage full-length 16S rRNA gene sequencing by the NANOPORE platform to depict compositional alterations in the COVID-19-associated oral microbiota at the species level and identify key specific microbial dysbiosis closely correlated with hospitalized patients infected with SARS-CoV-2 in two different regions of Anhui Province, China. Oropharyngeal swab specimens were collected starting on February 18, 2020.

Our study revealed obvious alterations in oral microbiota composition, which were characterized by several significant changes in microbial taxa and deviation in community structure, and identified three important bacterial signatures that might be influenced by SARS-CoV-2 infection. The decrease in commensal bacterial diversity has been suggested as a key symbol of dysbacteriosis in several diseases. However, the oral microbiota of mild COVID-19 patients in our research showed coherent decreased richness (Chao1 index, not significant) but increased Shannon biodiversity compared to the HCs, which showed inconformity to previous research (Iebba et al., 2021; Ma et al., 2021). Nonetheless, when we divided SARS-CoV-2 patients into two groups with higher and lower viral burdens, interestingly, a significantly increased trend of alpha richness (Chao1 index) and Shannon biodiversity, although not significant, was found in those patients with higher SARS-CoV-2 viral load, which was inconsistent with previous studies (Gao et al., 2021; Ma et al., 2021; Wu et al., 2021). These results indicated dysbiosis in the oropharyngeal bacteria of patients infected with SARS-CoV-2. Thus, we speculated that the taxa of oral bacteria enriched in mild COVID-19, such as *P. anaerobius* and *P. stutzeri*, may act as pathogens when enriched in the oral cavity or escaping to the gut when swallowing. For instance, convincing data demonstrated the detrimental role of elevated levels of *P. anaerobius* in contributing to colorectal carcinogenesis (Tsoi et al., 2017; Long et al., 2019), confirming that the oral cavity may be a source of pathogens that infect the gastrointestinal tract. Several studies have confirmed a potential causal link between the oral cavity and gut in the pathogenesis of infectious disease due to the profound interaction between the oral–gut microbiome in regulating host mucosal immunity by translocation (Cao, 2017; Lira-Junior and Bostrom, 2018; Read et al., 2021). Hence, the aforementioned results should be considered clinically significant because variations in the oropharyngeal microbiota of COVID-19 patients can be used as rapid and non-invasive bacterial biomarkers of dysbiosis of the gut microbiota or invasion of potential pathogens in the gut region that cannot be reflected by feces. *Campylobacter* and *P. stutzeri*, two enriched taxa in the oral cavity of COVID-19 patients, are reported as opportunistic pathogens for periodontitis progression, pneumonia, meningitis, and infective endocarditis (Ihara et al., 2003; Tasdelen Fisgin et al., 2004; Alwazzeh et al., 2020). Though the enrichment of *Campylobacter* in LCP patients was in agreement with the previous publication (Wu et al., 2021), that the majority of oral bacteria increased or decreased in COVID-19 patients in our work was shown to disagree with other reports. In addition, some increased bacteria were previously identified as bacteremia-associated bacteria, implying a potential association between oral dysbiosis and secondary bacterial infection in COVID-19 patients.

We identified several candidate driver taxa (e.g., *S. anginosus*, *S. alactolyticus*, and *P. stutzeri*) that played a key role in driving the changes in microbial correlation networks between COVID-19 patients and HCs. *S. anginosus*/*alactolyticus* has previously been shown to colonize the upper respiratory, digestive, and reproductive tracts. However, under certain stimuli, such as SARS-CoV-2, disordered *S. anginosus* directly induces non-invasive infections and causes invasive infections after entering normal sterile sites in the body, including the blood and serosal cavity, which eventually affect the tissues and organs of various systems of the body (Jiang et al., 2020; Vinciguerra et al., 2021). In addition, the results of the classification model identified three species as the most important signatures for representing SARS-CoV-2 infection from healthy individuals. Nevertheless, all 3 bacterial signatures were reduced in the patient group and offered alternatives for SARS-CoV-2 detection by quantifying the levels of *P. veronii*, *P. fragi*, and *S. yabuuchiae* by qPCR. Strikingly, we found a positive and reverse correlation between decreased taxa in SARS-CoV-2 patients with higher viral load and blood MCV, as well as MCHC. To some extent, it shows that the alteration of oral microbiota caused by virus infection affects the inflammatory indicators in the blood. These results suggest that viral infection can exacerbate host inflammation by affecting oral microorganisms that further influence blood circulation indicators.

Thus, the results of the present study provide novel clinical findings. First, the data reveal that the variation in the oropharyngeal microbiome may be used as a non-invasive biomarker aid in the diagnosis of patients with SARS-CoV-2 infection; second, we provide some evidence that major potential pathogens are associated with lung or gut coinfections in COVID-19 to guide antibiotic treatment of secondary bacterial infections in COVID-19; third, bacterial strains, such as *P. anaerobius*, identified in the present study require future studies to determine their roles in the pathogenicity of SARS-CoV-2 and in COVID-19 development; fourth, the data provide a compelling rationale suggesting that effective oral hygiene measures and promotions are necessary to reduce secondary infections, especially in patients with severe COVID-19.

Being attractive for the ease of sampling, as demonstrated in international projects such as HMP, oral swab sampling (touching tongue, palatum, and cheeks) would be a valid alternative to the current frequently used specimens (e.g., feces and blood) to assess the microbiota compositional differences in disease, giving reliable insights into subject susceptibility. There are limitations to our study: 1) the low number of subjects involved, especially the loss of severe SARS-CoV-2-infected patients; 2) the lack of shotgun metagenomics to ascertain genes and/or pathways; and 3) the inability to confirm whether the observed oral dysbiosis was already in action in COVID-19 patients when oral swab samples were taken. Despite these restrictions, our study leveraged full-length 16S rRNA gene sequencing technology to improve the observation of taxonomic variation at the species level. This research provides a hint at the importance of modulating the oral microbiota for the treatment or detection of COVID-19 symptoms.

## Conclusions

The present study might be the first to depict the alteration in the oral microbial composition of mild COVID-19 patients during hospitalization by nanopore technology based on the full-length 16S rRNA gene, despite the limited sample size. Nanopore sequencing would accelerate and aid the identification of maladjusted bacteria and would promote accuracy in taxonomic classification. In addition, the links between unbalanced oral microbiota and viral load in patients have denoted the potential of intervention in the prevention and treatment of SARS-CoV-2-infected patients based on the modulation of the oral microbiome.

## Data Availability Statement

The datasets generated and/or analysed during the current study are available from the corresponding author on reasonable request.

## Ethics Statement

The studies involving human participants were reviewed and approved by the Ethics Committee of Anhui Medical University (approval number: 20200291). The patients/participants provided their written informed consent to participate in this study.

## Author Contributions

S-HH and YS conceived and designed the experiments and revised the manuscript; M-ZHe performed the experiments, analyzed the data and wrote the manuscript; H-YG, PW, J-LY and Y-LG collected the samples; Y-LS and M-ZHa performed bioinformatic analysis and interpreted the data. All authors read and approved the final manuscript.

## Funding

This work was supported by the National Natural Science Foundation of China (81974306) and Emergency Research Project of Novel Coronavirus Infection of Anhui Province (202004a07020004 and 202004a07020002).

## Conflict of Interest

The authors declare that the research was conducted in the absence of any commercial or financial relationships that could be construed as a potential conflict of interest.

## Publisher’s Note

All claims expressed in this article are solely those of the authors and do not necessarily represent those of their affiliated organizations, or those of the publisher, the editors and the reviewers. Any product that may be evaluated in this article, or claim that may be made by its manufacturer, is not guaranteed or endorsed by the publisher.

## Abbreviations

COVID-19, coronavirus disease 2019; SARS-CoV, severe acute respiratory syndrome coronavirus 2; RT–PCR, reverse transcriptase–PCR; qPCR, quantitative real-time PCR; rRNA, ribosomal ribonucleic acid; Ct., cycle threshold; LCP, laboratory-confirmed SARS-CoV-2 infection patients; HCs, healthy individuals; SparCC, sparse correlations for compositional data; RBI, routine blood indicators.

## References

[B1] Alwazzeh M. J. Alkuwaiti F. A. Alqasim M. Alwarthan S. El-Ghoneimy Y. (2020). Infective Endocarditis Caused by Pseudomonas Stutzeri: A Case Report and Literature Review. Infect. Dis. Rep. 12 (3), 105–109. doi: 10.3390/idr12030020 33276629 PMC7768374

[B2] Bao L. Zhang C. Dong J. Zhao L. Li Y. Sun J. (2020). Oral Microbiome and SARS-CoV-2: Beware of Lung Co-Infection. Front. Microbiol. 11, 1840. doi: 10.3389/fmicb.2020.01840 32849438 PMC7411080

[B3] Benjamini Y. Hochberg Y. (1995). Controlling the False Discovery Rate: A Practical and Powerful Approach to Multiple Testing. J. R. Stat. Soc.: Ser. B (Methodol.) 57 (1), 289–300. doi: 10.1111/j.2517-6161.1995.tb02031.x

[B4] Cao X. (2017). Intestinal Inflammation Induced by Oral Bacteria. Science 358 (6361), 308–309. doi: 10.1126/science.aap9298 29051367

[B5] Cheng M. P. Papenburg J. Desjardins M. Kanjilal S. Quach C. Libman M.. (2020). Diagnostic Testing for Severe Acute Respiratory Syndrome-Related Coronavirus 2: A Narrative Review. Ann. Intern. Med. 172 (11), 726–734. doi: 10.7326/M20-1301 32282894 PMC7170415

[B6] Coronavirus W (2021). Dashboard | WHO Coronavirus (COVID-19) Dashboard With Vaccination Data.

[B7] Freire M. Nelson K. E. Edlund A. (2021). The Oral Host-Microbial Interactome: An Ecological Chronometer of Health? Trends Microbiol. 29 (6), 551–561. doi: 10.1016/j.tim.2020.11.004 33279381

[B8] Friedman J. Alm E. J. (2012). Inferring Correlation Networks From Genomic Survey Data. PloS Comput. Biol. 8 (9), e1002687. doi: 10.1371/journal.pcbi.1002687 23028285 PMC3447976

[B9] Gao M. Wang H. Luo H. Sun Y. Wang L. Ding S.. (2021). Characterization of the Human Oropharyngeal Microbiomes in SARS-CoV-2 Infection and Recovery Patients. Adv. Sci. (Weinh) 8 (20), 2102785. doi: 10.1002/advs.202102785 PMC8529429 34423593

[B10] Iebba V. Zanotta N. Campisciano G. Zerbato V. Di Bella S. Cason C.. (2021). Profiling of Oral Microbiota and Cytokines in COVID-19 Patients. Front. Microbiol. 12, 671813. doi: 10.3389/fmicb.2021.671813 34394024 PMC8361794

[B11] Ihara H. Miura T. Kato T. Ishihara K. Nakagawa T. Yamada S.. (2003). Detection of Campylobacter Rectus in Periodontitis Sites by Monoclonal Antibodies. J. Periodontal. Res. 38 (1), 64–72. doi: 10.1034/j.1600-0765.2003.01627.x 12558939

[B12] Jiang S. Li M. Fu T. Shan F. Jiang L. Shao Z. (2020). Clinical Characteristics of Infections Caused by Streptococcus Anginosus Group. Sci. Rep. 10 (1), 9032. doi: 10.1038/s41598-020-65977-z 32493976 PMC7270121

[B13] Jiao L. Li H. Xu J. Yang M. Ma C. Li J.. (2021). The Gastrointestinal Tract Is an Alternative Route for SARS-CoV-2 Infection in a Nonhuman Primate Model. Gastroenterology 160 (5), 1647–1661. doi: 10.1053/j.gastro.2020.12.001 33307034 PMC7725054

[B14] Kuntal B. K. Chandrakar P. Sadhu S. Mande S. S. (2019). ‘Netshift’: A Methodology for Understanding ‘Driver Microbes’ From Healthy and Disease Microbiome Datasets. ISME J. 13 (2), 442–454. doi: 10.1038/s41396-018-0291-x 30287886 PMC6331612

[B15] Lira-Junior R. Bostrom E. A. (2018). Oral-Gut Connection: One Step Closer to an Integrated View of the Gastrointestinal Tract? Mucosal Immunol. 11 (2), 316–318. doi: 10.1038/mi.2017.116 29297500

[B16] Li Y. Wang K. Zhang B. Tu Q. Yao Y. Cui B.. (2019). Salivary Mycobiome Dysbiosis and its Potential Impact on Bacteriome Shifts and Host Immunity in Oral Lichen Planus. Int. J. Oral. Sci. 11 (2), 13. doi: 10.1038/s41368-019-0045-2 31263096 PMC6802619

[B17] Long X. Wong C. C. Tong L. Chu E. S. H. Ho Szeto C. Go M. Y. Y.. (2019). Peptostreptococcus Anaerobius Promotes Colorectal Carcinogenesis and Modulates Tumour Immunity. Nat. Microbiol. 4 (12), 2319–2330. doi: 10.1038/s41564-019-0541-3 31501538

[B18] Mammen M. J. Scannapieco F. A. Sethi S. (2020). Oral-Lung Microbiome Interactions in Lung Diseases. Periodontol 83 (1), 234–241. doi: 10.1111/prd.12301 32385873

[B19] Ma S. Zhang F. Zhou F. Li H. Ge W. Gan R.. (2021). Metagenomic Analysis Reveals Oropharyngeal Microbiota Alterations in Patients With COVID-19. Signal Transduct. Target Ther. 6 (1), 191. doi: 10.1038/s41392-021-00614-3 33986253 PMC8116522

[B20] Netz R. R. Eaton W. A. (2020). Physics of Virus Transmission by Speaking Droplets. Proc. Natl. Acad. Sci. U. S. A. 117 (41), 25209–25211. doi: 10.1073/pnas.2011889117 32973098 PMC7568337

[B21] Read E. Curtis M. A. Neves J. F. (2021). The Role of Oral Bacteria in Inflammatory Bowel Disease. Nat. Rev. Gastroenterol. Hepatol. 18 (10), 731–742. doi: 10.1038/s41575-021-00488-4 34400822

[B22] Ren Z. Wang H. Cui G. Lu H. Wang L. Luo H.. (2021). Alterations in the Human Oral and Gut Microbiomes and Lipidomics in COVID-19. Gut 70 (7), 1253–1265. doi: 10.1136/gutjnl-2020-323826 33789966 PMC8042598

[B23] Su Y. C. F. Anderson D. E. Young B. E. Linster M. Zhu F. Jayakumar J.. (2020). Discovery and Genomic Characterization of a 382-Nucleotide Deletion in ORF7b and ORF8 During the Early Evolution of SARS-CoV-2. mBio 11 (4), e01610–20. doi: 10.1128/mBio.01610-20 32694143 PMC7374062

[B24] Tasdelen Fisgin N. Acuner I. C. Coban A. Y. Fisgin T. Birinci A. Durupinar B. (2004). Meningitis Due to Pseudomonas Stutzeri: A Case Report. Mikrobiyol. Bul. 38 (3), 261–264. 15490847

[B25] Tsoi H. Chu E. S. H. Zhang X. Sheng J. Nakatsu G. Ng S. C.. (2017). Peptostreptococcus Anaerobius Induces Intracellular Cholesterol Biosynthesis in Colon Cells to Induce Proliferation and Causes Dysplasia in Mice. Gastroenterology 152 (6), 1419–1433 e1415. doi: 10.1053/j.gastro.2017.01.009 28126350

[B26] Vinciguerra M. Santamaria V. Romiti S. D’Abramo M. Toto G. De Bellis A.. (2021). Case Report: Streptococcus Alactolyticus as a Rare Pathogen of Mitral Endocarditis. Front. Cardiovasc. Med. 8, 648213. doi: 10.3389/fcvm.2021.648213 33996945 PMC8116484

[B27] Wolfel R. Corman V. M. Guggemos W. Seilmaier M. Zange S. Muller M. A.. (2020). Virological Assessment of Hospitalized Patients With COVID-2019. Nature 581 (7809), 465–469. doi: 10.1038/s41586-020-2196-x 32235945

[B28] Wu Y. Cheng X. Jiang G. Tang H. Ming S. Tang L.. (2021). Altered Oral and Gut Microbiota and its Association With SARS-CoV-2 Viral Load in COVID-19 Patients During Hospitalization. NPJ Biofilms Microbiomes 7 (1), 61. doi: 10.1038/s41522-021-00232-5 34294722 PMC8298611

[B29] Xiang Z. Koo H. Chen Q. Zhou X. Liu Y. Simon-Soro A. (2020). Potential Implications of SARS-CoV-2 Oral Infection in the Host Microbiota. J. Oral. Microbiol. 13 (1), 1853451. doi: 10.1080/20002297.2020.1853451 33312449 PMC7711743

[B30] Yeoh Y. K. Zuo T. Lui G. C. Zhang F. Liu Q. Li A. Y.. (2021). Gut Microbiota Composition Reflects Disease Severity and Dysfunctional Immune Responses in Patients With COVID-19. Gut 70 (4), 698–706. doi: 10.1136/gutjnl-2020-323020 33431578 PMC7804842

[B31] Zhang H. Ai J. W. Yang W. Zhou X. He F. Xie S.. (2021). Metatranscriptomic Characterization of Coronavirus Disease 2019 Identified a Host Transcriptional Classifier Associated With Immune Signaling. Clin. Infect. Dis. 73 (3), 376–385. doi: 10.1093/cid/ciaa663 32463434 PMC7314197

[B32] Zhou P. Yang X. L. Wang X. G. Hu B. Zhang L. Zhang W.. (2020). A Pneumonia Outbreak Associated With a New Coronavirus of Probable Bat Origin. Nature 579 (7798), 270–273. doi: 10.1038/s41586-020-2012-7 32015507 PMC7095418

[B33] Zhu N. Zhang D. Wang W. Li X. Yang B. Song J.. (2020). A Novel Coronavirus From Patients With Pneumonia in China, 2019. N. Engl. J. Med. 382 (8), 727–733. doi: 10.1056/NEJMoa2001017 31978945 PMC7092803

[B34] Zuo T. Liu Q. Zhang F. Lui G. C. Tso E. Y. Yeoh Y. K.. (2021). Depicting SARS-CoV-2 Faecal Viral Activity in Association With Gut Microbiota Composition in Patients With COVID-19. Gut 70 (2), 276–284. doi: 10.1136/gutjnl-2020-322294 32690600 PMC7385744

[B35] Zuo T. Zhang F. Lui G. C. Y. Yeoh Y. K. Li A. Y. L. Zhan H.. (2020). Alterations in Gut Microbiota of Patients With COVID-19 During Time of Hospitalization. Gastroenterology 159 (3), 944–955 e948. doi: 10.1053/j.gastro.2020.05.048 32442562 PMC7237927

