# Supplementary material for: Characterization of Altered Oropharyngeal Microbiota in Hospitalized Patients With Mild SARS-CoV-2 Infection
Source: Front Cell Infect Microbiol. 2022 Mar 15;12:824578. doi: 10.3389/fcimb.2022.824578 (PMC8965315; doi:10.3389/fcimb.2022.824578)
Supplement: Supplementary file 1 [file DataSheet_1.docx]

**Characterization of altered oropharyngeal microbiota in hospitalized patients with mild SARS-CoV-2 infection**

Yong-Lin Shi^†3^, Mao-Zhang He^†1^, Mao-Zhen Han^†2^, Hong-Ya Gui^1^, Peng Wang^3^, Jun-Ling Yu^3^, Ying-Lu Ge^3^, Yong Sun^*3^, Sheng-Hai Huang^*1,2^

*To whom correspondence should be addressed

^1^Department of Microbiology, The Key Laboratory of Microbiology and Parasitology of Anhui Province, The Key Laboratory of Zoonoses of High Institutions in Anhui, School of Basic Medical Sciences, Anhui Medical University, No. 81 Meishan Road, Hefei 230022, P.R. China

^2^School of Life Sciences, Anhui Medical University, No. 81 Meishan Road, Hefei 230022, P.R. China

^3^Anhui Provincial Center for Disease Control and Prevention, 12560, Fanhua Avenue, Hefei, P.R. China

**Supporting information**

Supplementary Figure 1. Oral microbial diversity analysis between higher and lower SARS-CoV-2 viral load patients.

Supplementary Figure 2. The “driver” taxa responsible for the change of microbial correlations between Health controls and Mild SARS-CoV-2 infection patients

Supplementary Table 1. Characteristics of microbial correlation networks associated with different groups.


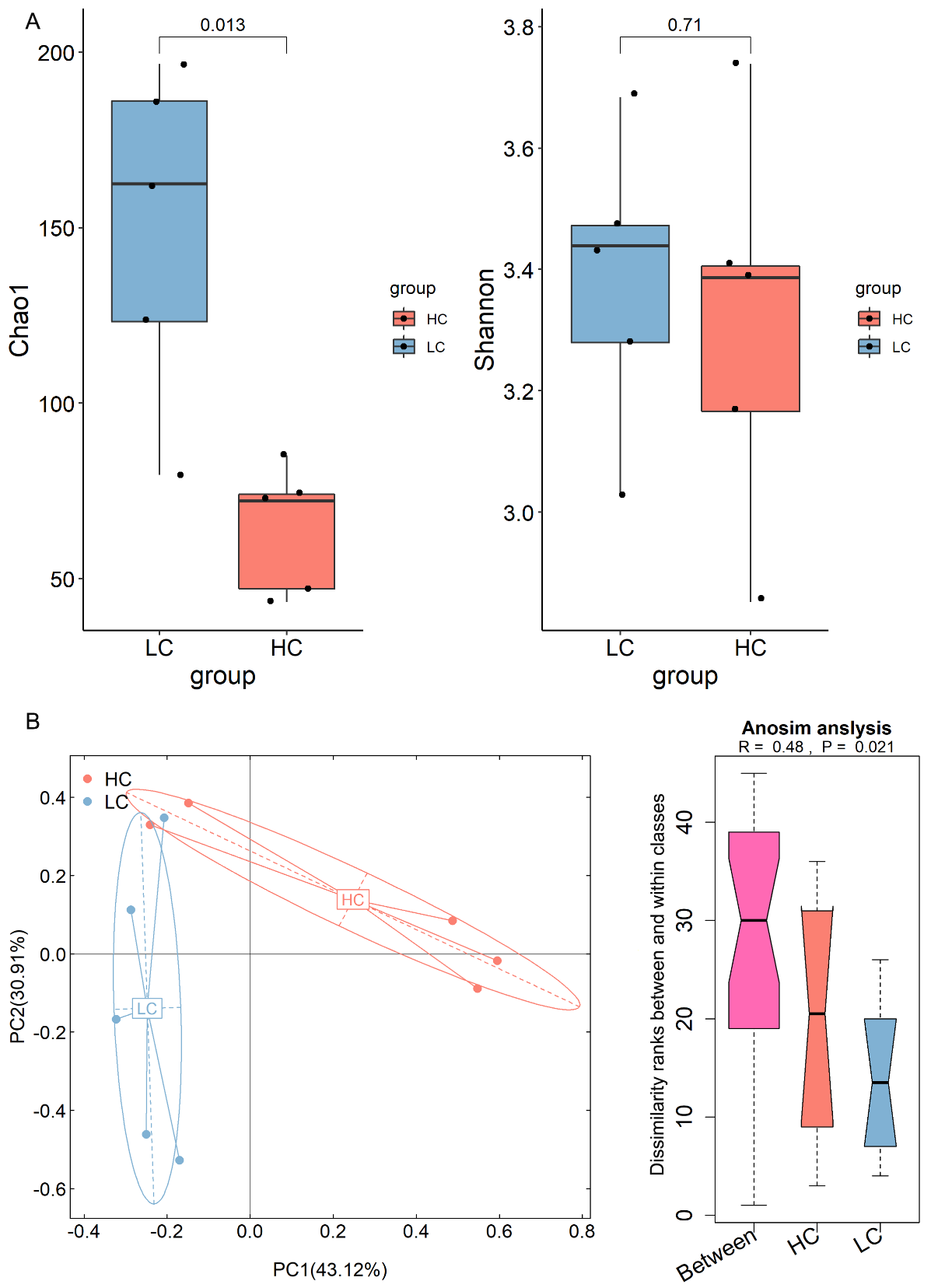


**Supplementary Figure 1. Oral microbial diversity analysis between higher and lower SARS-CoV-2 viral load patients.** (A) Species diversity differences between LCP and HC group, patients with higher SARS-CoV-2 viral load showed decreased microbial diversity. (B) The PCoA plot based on species level showed that the oral taxonomic composition was conspicuously different between the groups, which corroborated by Anosim analysis in the right panel.


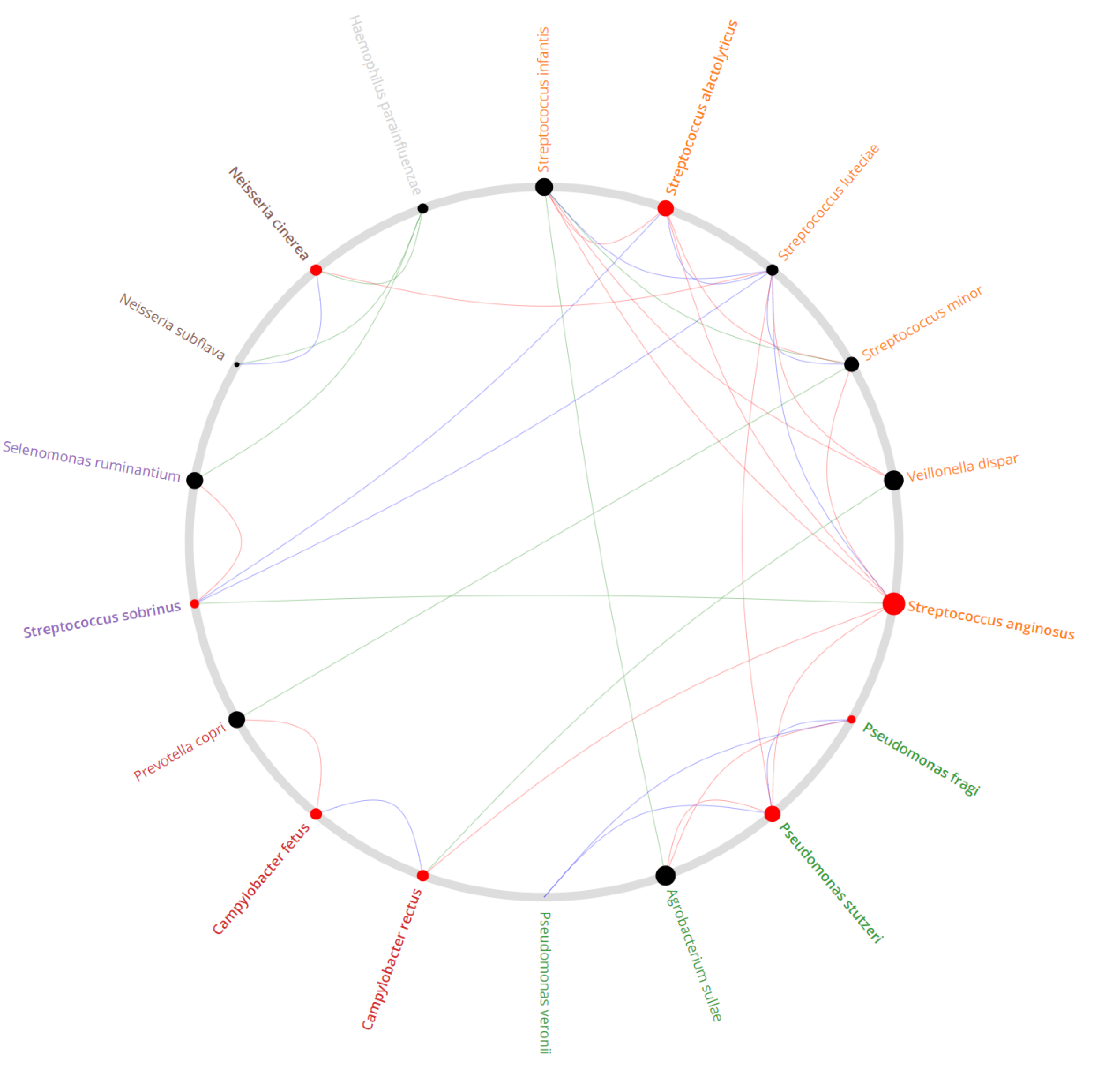


**Supplementary Figure 2. The “driver” taxa responsible for the change of microbial correlations between Health controls and Mild SARS-CoV-2 infection patients.** Node sizes are proportional to their scaled neighbor shift (NESH) score (i.e., a score identifying important microbial taxa of microbial association networks). A node is colored red if it is a “driver” node/taxon: its betweenness increases when comparing microbial correlation networks of LCP with that of HC. All taxa belonging to same community (common sub-network) are randomly assigned a color to their labels. Red (or green) edges represent microbial correlations that are only present in the LCP (or HC) network, respectively. Blue edges present common microbial correlations that are present in both networks.

**Supplementary Table 1.** **Characteristics of microbial correlation networks associated with different groups.** In order to quantify the difference of the network structure, we calculated the number of nodes, number of edges, average degree (the average number of connections per node), betweenness centrality (measure the extent to which a vertex lies on paths between other vertices), closeness (measure measures the mean distance from a vertex to other vertices).

| **Groups** | **Average degree** | **Closeness** | **Edges** | **Betweenness**  **centrality** | **Nodes** |
| --- | --- | --- | --- | --- | --- |
| **Health** | 1.867 | 0.001 | 28 | 1.8 | 30 |
| **Mild** | 3.12 | 0.006 | 39 | 14.48 | 25 |
